# Case report: Male genital system, soft tissue and myocardial metastases in a patient with exon 11-mutated GIST of unknown origin

**DOI:** 10.3389/fonc.2024.1450889

**Published:** 2024-09-03

**Authors:** Michele Rota, Federico Sganzerla, Michele Zuffante, Andrea Mafficini, Michele Pavarana, Michele Milella

**Affiliations:** ^1^ Section of Innovation Biomedicine - Oncology Area, Department of Engineering for Innovation Medicine, University of Verona and Verona University and Hospital Trust, Verona, Italy; ^2^ Nuclear Medicine Unit, Integrated University Hospital of Verona, Verona, Italy; ^3^ Department of Engineering for Innovation Medicine, University of Verona and Verona University and Hospital Trust, Verona, Italy; ^4^ Applied Research on Cancer (ARC)-Net Centre for Applied Research on Cancer, University and Hospital Trust of Verona, Verona, Italy

**Keywords:** GIST, metastases, exon 11 mutation, imatinib, case report

## Abstract

Gastrointestinal stromal tumors (GISTs) are the most frequent mesenchymal tumors of the gastrointestinal tract, usually arising in the stomach or in the small bowel. Most GISTs are diagnosed early due to the presence of symptoms (e.g., abdominal discomfort/pain, anemia, etc.); at times, diagnosis could be incidental (e.g., ultrasound or endoscopic examinations performed for other reasons, surgical intervention for a different disease, etc.). Diagnosis occurs when the tumor is already metastatic in 10-20% of cases. The most common metastatic sites are liver, peritoneum, and loco-regional lymph nodes. Here, we present the case of a male patient with an atypical presentation of disease: as a matter of fact, during his oncological history, he developed metastases in unlikely sites, such as penis, scrotum, myocardium, and soft tissues.

## Introduction

Gastrointestinal Stromal Tumors (GISTs) are malignant mesenchymal tumors derived from interstitial cells of Cajal or their precursors with an annual incidence of 10-15 cases per million. The pathogenesis is associated in 95% of cases with mutations of tyrosine kinase receptor gene *KIT* (CD117) or Platelet Derived Growth Factor Receptor-A (*PDGFR-A*), but genomic alterations such as Neurotrophic Tyrosine Receptor Kinase (*NTRK*) rearrangements and B-Rapidly Accelerated Fibrosarcoma (*BRAF*) mutations could be rarely found.

GISTs belong to the soft tissue sarcoma family and are the most common ones in the gastrointestinal tract. The prevalent primary sites are stomach (60-70%) and small intestine (20-30%), whereas less frequent sites are colon and esophagus (1-5%) (1).

Detecting GISTs can sometimes be challenging, as they may be entirely asymptomatic or present with nonspecific signs and symptoms, such as vague abdominal discomfort or anemia caused by chronic bleeding that can also lead to positive fecal occult blood test. In other cases, GISTs may present acutely with massive bleeding and hemodynamic instability (2).

Tyrosine Kinase Inhibitors (TKIs) represents the backbone of GIST treatment in the metastatic setting (3); in particular, patients’ survival rates have improved significantly since the approval of imatinib (4, 5). Most of GISTs show sensitivity to imatinib, in particular when a mutation of the exon 11 of *KIT* is detected, and a dose of 400 mg/d is recommended. When a mutation of the exon 9 of the *KIT* gene occurs, a double dose of imatinib (800 mg/d) is indicated (6).

At progression, sunitinib (50 mg 4 weeks on/2 weeks off or 37.5 mg continuously) is the standard second line of therapy (7). At further failure, regorafenib represents another approved treatment (8). In May 2020, FDA approved ripretinib for patients with metastatic GISTs that had progressed to three or more TKIs, including imatinib (9).

Secondary *KIT* mutations represent the most frequent mechanism of acquired resistance to imatinib and other TKIs, leading to progressive disease and the need to change the therapeutic strategy (10).

In rare cases, *NTRK* rearrangements and *BRAF* mutations can be discovered, which could be sensitive respectively to NTRK inhibitors (such as entrectinib or larotrectinib) and BRAF inhibitors (11).

The most common sites of metastasizing are liver (50-60%), peritoneum (30-40%) and loco-regional lymph nodes (12). Nevertheless, in some cases GISTs can metastasize in less common sites like bone, bone marrow, intracranial area, skin and subcutaneous tissue, skeletal muscle, orbit, pancreas, spleen, heart, gonads, kidneys, and bladder.

Here, we present an unusual case of GIST, arising in a male patient, and presenting with a high burden of disease at baseline and the development of multiple metastases in uncommon sites.

## Case report

In March 2019, a 57-year-old man in good clinical conditions (Eastern Cooperative Oncology Group Performance Status 0) and without significant comorbidities or familiarity for oncological diseases underwent ultrasound examination of the abdomen for abdominal discomfort and night sweating over the past three months; furthermore, palpable masses localized in the right hypochondrium and epigastrium had become evident. Ultrasound examination showed multiple expansive lesions in the right hepatic lobe (the biggest one measuring 14 cm in the longest diameter) and suspected peritoneal localizations. Computed Tomography (CT) scan confirmed both hepatic involvement (biggest lesion: 16x14 cm in S6) and peritoneal involvement (biggest lesion: 16x11cm, close to the descending colon). Eventually, a Positron Emission Tomography (PET) examination was performed, with uptake in the lesions highlighted at the CT scan. No primitive tumor was found.

Liver biopsy demonstrated liver metastasis of spindle cell GIST (IHC: CD117+, DOG1+, MDM2+, Ki67 20%). A mutation in exon 11 of the *KIT* gene was detected (p.Thr574_Asp579dup), whereas *BRAF, PDGFR-A, PDGFR-B, SDHA* genes were all wild-type and immunohistochemistry analysis for pan-TRK was negative.

**Figure 1:**
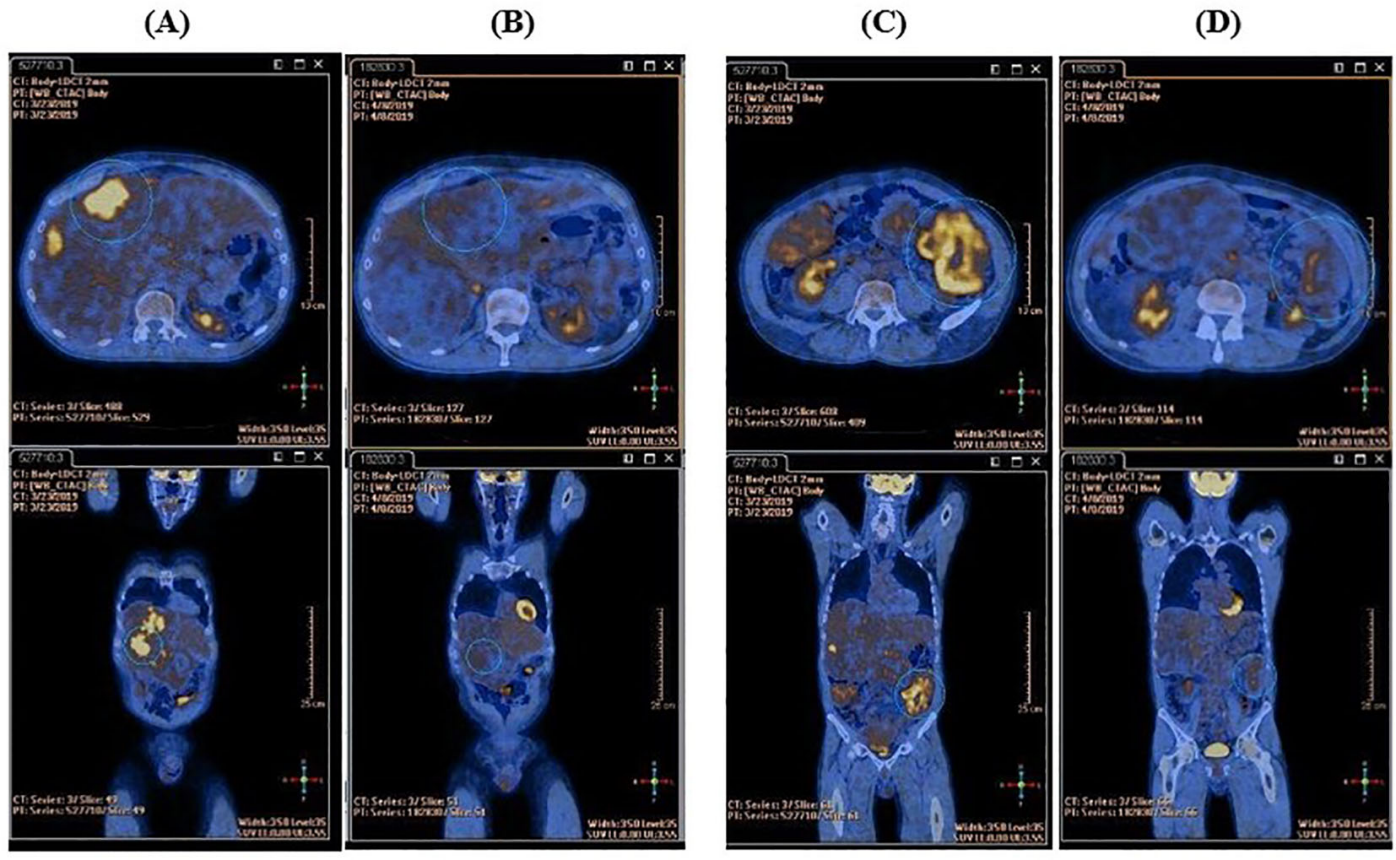
Comparison between the basal FDG-PET and the FDG-PET performed after two weeks of imatinib therapy. **(A)** High tracker uptake in liver metastasis at baseline. **(B)** Complete metabolic response in liver metastasis after two weeks of imatinib therapy. **(C)** High tracker uptake in peritoneal metastasis at baseline. **(D)** Near-complete metabolic response in peritoneal metastasis after two weeks of imatinib therapy.

In March 2019, first-line therapy with imatinib (400 mg/d) was started. PET examination, performed two weeks after treatment start, demonstrated near-complete metabolic response with absence of FDG uptake in the known sites of disease (**Figure 1**).

Best response at CT scan-based restaging was partial response (PR); treatment was well-tolerated.

Unfortunately, in August 2021, CT scan demonstrated hepatic and peritoneal disease progression (PD); imatinib’s dose was escalated to 800 mg/d, with further hepatic PD. Second-line therapy with sunitinib was then started in December 2021.

**Figure 2:**
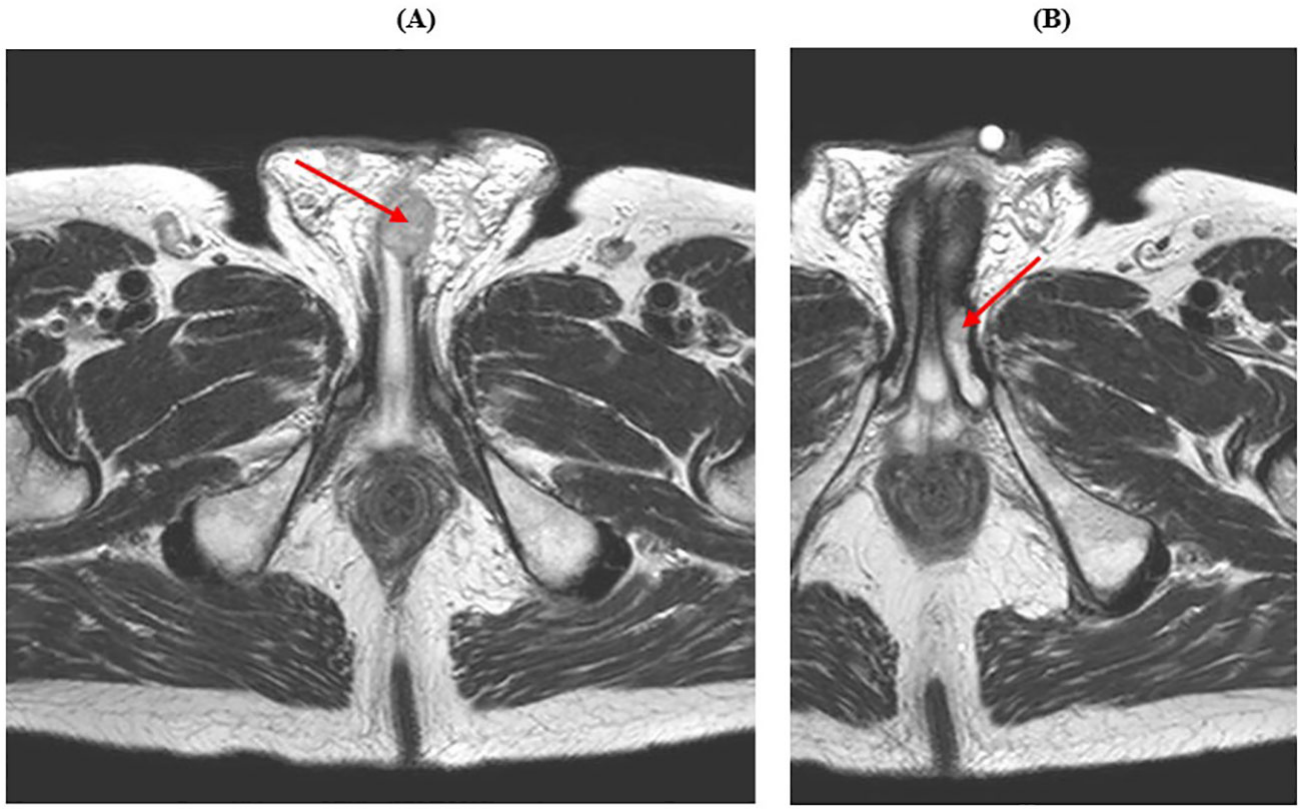
Pelvic MRI showing male genital system metastases. **(A)** The red arrow indicates the lesion in the corpus spongiosum. **(B)** The red arrow indicates the lesion in the corpus cavernosum.

In January 2022, the patient reported urinary discomfort and swelling at the base of the penis; pelvic Magnetic Resonance Imaging (MRI) demonstrated a 16x26x13 mm nodule in the corpus spongiosum, which caused compression on the urethra “ab estrinseco”, and a 7x9x10 mm lesion in the corpus cavernosum (**Figure 2**).

In February 2022, the patient was admitted to the Urology Department and the corpus spongiosum lesion was removed; histological examination was compatible with the diagnosis of GIST (IHC: CD117+, DOG1+) and the margins of resection were free of neoplasia (R0). The urinary problem was resolved and the sunitinib therapy was restarted.

CT scan performed in April 2022 showed further hepatic PD and third-line therapy with regorafenib was started. Best response was stable disease (SD), but the treatment was poorly tolerated, due to skin toxicity and hypertension.

In October 2022 MRI showed penile PD and appearance of a lymph node conglomerate in the right inguinal region; rechallenge with imatinib (400 mg/d) was started.

In February 2023 further penile PD, causing urinary discomfort, was documented by MRI together with the appearance of a lesion in the right gluteus medius and multiple scrotal nodules (biggest lesion: 38x32 mm); two scrotal nodules were removed in May 2023 and histological examination confirmed the diagnosis of GIST metastases, with again a R0 resection.

**Figure 3:**
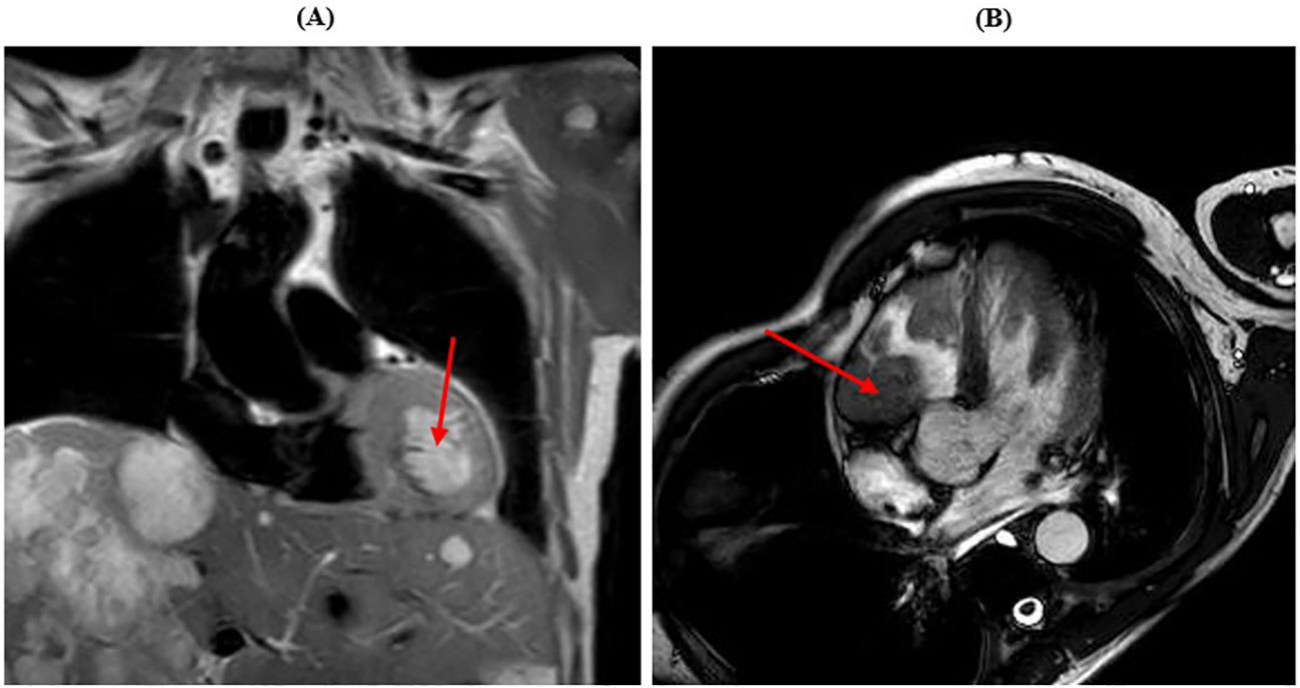
Cardiac MRI showing myocardial metastases. **(A)** The red arrow shows the lesion in the left ventricle. **(B)** The red arrow shows the lesion in the right ventricle.

Shortly thereafter, following an episode of amaurosis fugax, echocardiogram was performed, with the evidence of two right and left ventricular masses, with conserved left ventricular function. Cardiac MRI confirmed a mass near the left ventricular apex (4 cm), a mass in the right ventricle under the tricuspid valve (5 cm) and another mass in the right atrium near the atrial septum (3 cm), with imaging strongly suggestive for GIST metastases (**Figure 3**).

The patient eventually died, due to a cardiac arrest while sleeping, in July 2023.

**Figure 4:**
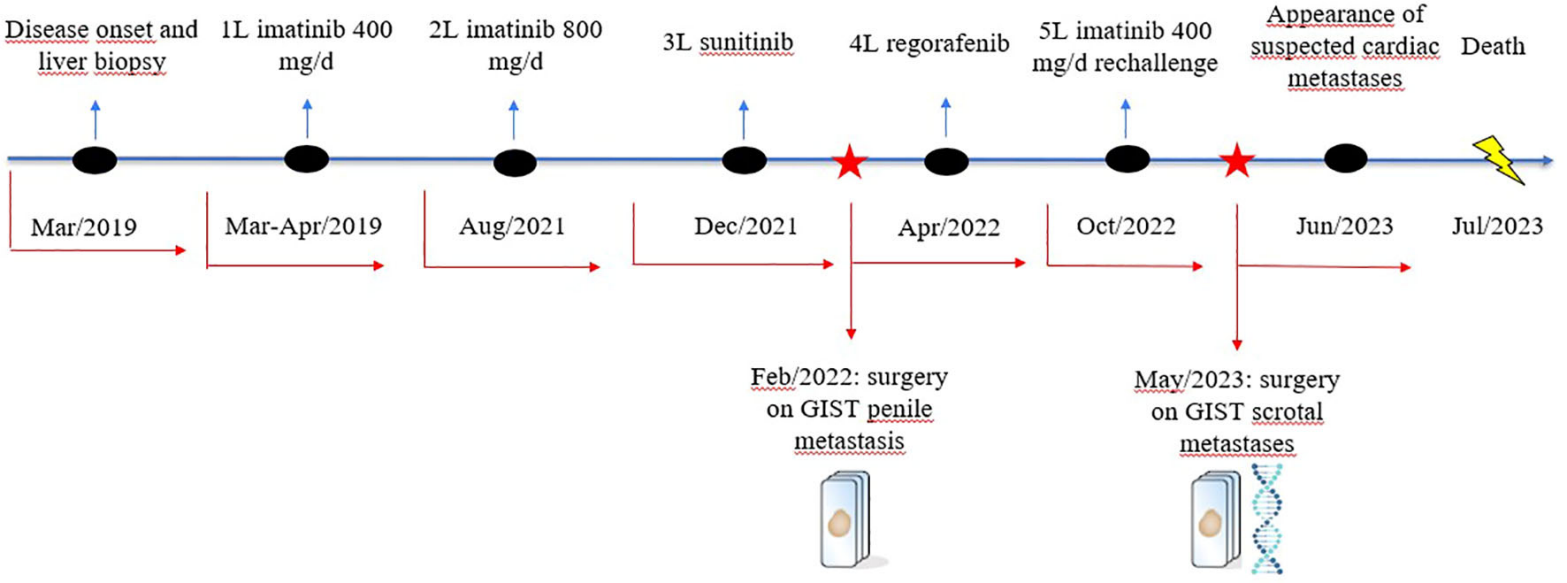
Schematic representation of the patient’s treatment history.

To sum up, in the (**Figure 4**) is illustrated a schematic representation of the patient’s treatment history.

## Discussion

The unicity of the case presented here is related to the atypical disease behavior. GISTs most frequently metastasize to the liver and peritoneum and very infrequently outside the abdomen. A recent review regarding the rare metastatic localization of GIST showed that cardiac and soft tissue metastases are uncommon (1% of cases), but male genital system metastases are even more infrequent (< 1% of cases) (13). To the best of our knowledge, this is the first report of a patient with the concomitant presence of these three unusual localizations. While in this case the high disease burden at presentation may have favored widespread dissemination to unusual localizations, the reasons why sometimes GISTs metastasize to these rare sites remain unclear.

The specific mutation of the *KIT* gene (p.Thr574_Asp579dup) is an in-frame insertion resulting in a duplication of a stretch of 6 aminoacids located in the juxtamembrane domain of the protein. At present, it is not surveyed and functionally validated, but it is considered to be likely oncogenic and druggable by FDA- and EMA-approved imatinib (AMP tier 1, ESCAT tier 1) (14); only a few GISTs cases exhibiting this specific mutation have been reported in the literature (15). Although little is known on the biology deriving from this specific mutation, the clinical behavior observed in the case reported here is not routinely expected from GISTs harboring exon 11 mutations of the KIT gene: this subtype of tumors is frequently associated with an indolent course and better prognosis, while exon 9-mutated GISTs occur more frequently in extra-gastric sites, metastasize to unusual locations, and show a more aggressive and therapy-resistant course (16).

The duration of response to imatinib in this case is in line with expectations from registration studies (median time to treatment failure in responding patients: 122 weeks, 95% CI 106-147 weeks) (17), which is notable considering the “bulky” onset of disease, which is associated with a poorer prognosis. Early FDG-PET evaluation after treatment start showed rapid decreases of the Standardized Uptake Value (SUV), for the objective clinical response and satisfactory outcome subsequently observed, again in line with previous literature (18).

Molecular evolution of the disease was assessed by Next Generation Sequencing (NGS) analysis (Myriapod NGS Cancer Panel DNA-based, Illumina Platform, Diatech Pharmacogenetics) performed on one of the scrotal metastases removed in May 2023, at the time of the fourth progression (after imatinib, sunitinib, regorafenib, and imatinib rechallenge). Alongside with the same exon 11 mutation detected at baseline, we found another exon 17 *KIT* alteration (p.D820Y). As discussed above, secondary *KIT* mutations are a frequent mechanism of acquired resistance in patients pre-treated with either imatinib or other TKIs (10) and exon 17 mutations (including p.D820Y) seem to be the most frequent mechanisms of resistance to sunitinib (19).

Besides unusual presentation and uncommon sites of metastatic spread, another peculiar aspect may be of interest in this case: two of these unusual localizations (penile localizations to corpus spongiosum and scrotal lesions) were treated by surgical removal, based on the appearance of symptoms (in February 2022 and May 2023, respectively). In the metastatic setting, surgery is not routinely recommended for GISTs; however, in our case, surgery of progressing lesions has certainly improved patient’s quality of life and psychological balance and may have impacted on long-term outcome, for example by avoiding potential urinary obstruction and the need for more invasive interventions during second-line sunitinib.

## Conclusion

This case report shows an unusual presentation and clinical behavior of GIST, only partially explained by the specific mutational landscape and molecular evolution; in that respect, extended molecular profiling at presentation and non-invasive molecular monitoring could be important tools to guarantee better treatments for patients. Moreover, multidisciplinary integration with surgery and/or local ablative treatments may be employed to treat oligoprogression and maintain better quality of life.

## Data Availability

The raw data supporting the conclusions of this article will be made available by the authors, without undue reservation.
